# Dose-Response Relationship between Antimicrobial Drugs and Livestock-Associated MRSA in Pig Farming^1^

**DOI:** 10.3201/eid2106.140706

**Published:** 2015-06

**Authors:** Alejandro Dorado-García, Wietske Dohmen, Marian E.H. Bos, Koen M. Verstappen, Manon Houben, Jaap A. Wagenaar, Dick J.J. Heederik

**Affiliations:** Utrecht University, Utrecht, the Netherlands (A. Dorado-García, W. Dohmen, M.E.H. Bos, K.M. Verstappen, J.A. Wagenaar, D.J.J. Heederik);; Wageningen UR, Lelystad, the Netherlands (J.A. Wagenaar); PorQ BV, Son, the Netherlands (M. Houben).

**Keywords:** methicillin-resistant *Staphylococcus aureus*, antibacterial drug resistance, pigs, MRSA, zoonoses, risk reduction, antimicrobial resistance, bacteria

## Abstract

The farming community can be a vehicle for introduction of livestock-associated methicillin-resistant *Staphylococcus aureus* (LA-MRSA) in hospitals. During 2011–2013, an 18-month longitudinal study aimed at reducing the prevalence of LA-MRSA was conducted on 36 pig farms in the Netherlands. Evaluations every 6 months showed a slight decrease in MRSA prevalence in animals and a stable prevalence in farmers and family members. Antimicrobial use, expressed as defined daily dosages per animal per year, decreased 44% during the study period and was associated with declining MRSA prevalence in pigs. MRSA carriage in animals was substantially higher at farms using cephalosporins. Antimicrobial use remained strongly associated with LA-MRSA in humans regardless of the level of animal contact. A risk factor analysis outlined potential future interventions for LA-MRSA control. These results should encourage animal and public health authorities to maintain their efforts in reducing antimicrobial use in livestock and ask for future controlled intervention studies.

## Abstract 2

Decreasing antimicrobial use can lower MRSA prevalence in pigs and subsequently in humans.

In 2005, sequence type (ST) 398 of methicillin-resistant *Staphylococcus aureus* (MRSA) emerged in Europe with proven transmission between pigs and humans (*1*,*2*). Since then, pigs, veal calves, and (to a lesser extent) poultry were increasingly found to harbor livestock-associated MRSA (LA-MRSA) (*3*).

ST398 is widely spread across Europe, and ≈70% of pig farms in the Netherlands test positive (*4*). After transfer to humans, it can be introduced into hospitals and the community (*5*–*8*). In 2011, ST398 accounted for 39% of all new MRSA detected through screening of patients in the Netherlands (*9*).

To our knowledge, no intervention studies have been undertaken to assess the efficacy of MRSA-reducing measures on farms. Trade of animals is a major risk factor for introducing MRSA into a negative herd (*10*–*12*). Larger herds have been associated with higher antimicrobial use (*4*). Antimicrobial use could not be identified as a clear determinant for MRSA (*4*). Transmission dynamics within herds vary by animals’ ages and phase of production, potentially leading to endemicity (*13*).

In 2006, the European Union banned the use of antimicrobial drugs as growth promoters. In the Netherlands the most noticeable change started in 2010, when the government set objectives for a 50% reduction in antimicrobial use by 2013 and 70% by 2015, compared with 2009. This policy was combined with benchmarking of farms, and later veterinarians, to identify persistently high users of antimicrobial drugs (*14*). As part of this national program, farm treatment and health plans have to be drafted and reviewed annually (*15*), which has resulted in an almost 60% reduction for the major livestock industry sectors (*16*,*17*). Against the background of nationwide reduction of antimicrobial use, during 2011–2013, we evaluated MRSA carriage changes in pigs and humans and study the effect of introduction of an additional range of preventive measures on MRSA carriage in animals, and humans living and/or working on the farms.

## Materials and Methods

### Study Design, Sample Collection, and Laboratory Analysis

Thirty-six pig farms were enrolled in and completed the study; 15 were recruited from farmer cooperatives in the Netherlands, 20 were recruited by veterinarians in the cooperatives, and 1 was recruited by a farm health consultant. Farms were visited at the start of the study during March–September 2011. A questionnaire was completed during a walk-through survey with the farm veterinarian. The questionnaire contained items on farm characteristics, biosecurity, animal management and hygiene practices (Technical Appendix Table 1). Then, tailor-made interventions were developed with the farmer for each farm to be implemented from the beginning of the study. Interventions focused on 1) further reducing antimicrobial use, 2) improving personnel and farm hygiene, and 3) changing animal contact structures.

Each farm was assessed 4 times during the 18-month period (6-month intervals). At each sampling time, the farm questionnaire was filled out again to monitor changes. Human participants completed another questionnaire (Technical Appendix Table 2) focused on tasks performed, animal contact, and individual health status. Dry cotton nasal swabs (Copan, Brescia, Italy) were used to obtain samples from humans and animals. Persons self-sampled their nostrils, and veterinarians swabbed both anterior nares of 60 pigs per farm. Animal swab samples were analyzed in 10 pools of 6 animals. Each pool comprised pigs of the same age group in the same pen (suckling piglets, weaned piglets, gilts, sows, and finishing pigs). All animal and human samples were sent by courier to the Infectious Diseases and Immunology Department (Faculty of Veterinary Medicine, Utrecht University, Utrecht, the Netherlands). The Medical Ethical Committee of the University Medical Centre Utrecht approved the study protocol, and all participants gave written informed consent.

Swab samples were pre-enriched in Mueller Hinton broth, followed by selective enrichment with ceftizoxime and aztreonam and culture on Brilliance MRSA agar (Oxoid, Badhoevedorp, the Netherlands) (*18*). Suspected colonies were subcultured on Columbia agar with sheep blood (Oxoid) and confirmed by using real-time PCR targeting *mecA*, *femA*, *nuc*, and C01 genes (*19*,*20*).

### Farm Types

We classified production types as farrowing and farrow-to-finish. Farrowing farms did not produce fatteners and delivered growers (25 kg) to finishing farms (with the exception of 1 farm that delivered gilts for farrowing). Farrow-to-finish farms integrated farrowing and finishing production and delivered fattening pigs to the abattoir. A farm was defined as open when it received external supplies of gilts >1 time per year from at least 1 supplier and as closed when gilts were not supplied externally.

### Data on Antimicrobial Use

In the Netherlands, all antimicrobial drug deliveries to each farm are compiled in national databases. Owners of the study farms gave written consent for retrieval of these antimicrobial use data over a 2-year period. Antimicrobial use was expressed as defined daily dosages per animal per year (DDDA/Y) per farm for the 4 periods preceding each sampling time. The DDDA/Y is a standard weighted measure indicating the number of days of antimicrobial drug use per year for an average animal on the farm. A more detailed description of the calculation of DDDA/Y has been described (*14*,*16*).

### Data Analysis

We conducted all statistical analyses in SAS software version 9.2 (SAS Institute Inc., Cary, NC, USA). We explored changes in MRSA carriage in animals and humans and antimicrobial use over time using simple descriptive statistics. DDDA/Y was log_2_ transformed because of its right-skewed distribution. A total of 134 variables in the farm questionnaire and 59 in the human questionnaire were selected for longitudinal analysis together with antimicrobial use (criteria of <10% missing values and <10% of farms in each category). Odds ratios (ORs) for MRSA positivity in a pig or a human sample in the presence or absence of a determinant were obtained by using random intercept generalized linear mixed models (PROC GLIMMIX; SAS Institute, Inc.). Only associations from the selected variables with p<0.10 in pigs (adjusting for age group of the pool) and p<0.20 in humans (adjusting for hours worked on the farm) were presented. Goodness-of-fit of the models was described by using −2 log residual pseudo-likelihood estimation, and model assumptions were checked with diagnostic plots. Generalized additive mixed modeling (gamm4 package in R 3.0.2; R Foundation for Statistical Computing, Vienna, Austria) was used to assess the shape of the relationship between antimicrobial use and MRSA in human and animals.

## Results

The number of farms was unequally distributed by type of farm (Table 1). Characteristics among persons from different farm types did not differ significantly (Table 2). All MRSA isolated from animals and humans was ST398.

**Table 1:** Characteristics of farms in a study of the dose–response relationship between antimicrobial drug use and livestock-associated methicillin-resistant *Staphylococcus aureus* in pig farming, the Netherlands, 2011–2013

Type of farm*	No. farms	Median no. (interquartile range)	
		Sows	Fatteners
All	36	350 (270–550)	773 (0–1,950)
Open	22	337 (300–500)	500 (0–1,300)
Farrowing†	9	533 (350–800)	0
Farrow-to finish	13	314 (242–380)	1,100 (600–2,010)
Closed	14	407 (232–698)	1,400 (450–2,725)
Farrowing†	3	439 (239–905)	0
Farrow-to finish	11	367 (200–673)	1,892 (1,025–2,950)

*Farms were defined as open when they received external supplies of gilts ≥1 time per year from at least 1 supplier and as closed when they received no external supply of gilts. †No fattening pigs present.

**Table 2:** Characteristics of persons followed during the entire period of a study of the dose–response relationship between antimicrobial drug use and livestock-associated methicillin-resistant *Staphylococcus aureus* in pig farming, the Netherlands, 2011–2013*

Characteristic	Total study population	Farmers, employees	Partners	Children
Age, y (SD)	33.0 (17.8)	44.0 (13.6)	45.2 (8.9)	14.4 (5.6)
Mean time worked, h (SD)	21.8 (25.2)	46.0 (19.9)	10.1 (14.0)	2.2 (6.6)
Total no.	158	66	32	60
Sex				
M	91	58	0	33
F	67	8	32	27
Open farm	91	34	17	40
Farrowing†	26	11	5	10
Farrow-to finish	65	23	12	30
Closed farm	67	32	15	20
Farrowing†	14	8	3	3
Farrow-to finish	53	24	12	17

*Farms were defined as open when they received external supplies of gilts ≥1 time per year from at least 1 supplier and as closed when they received no external supply of gilts. †No fattening pigs present.

### Antimicrobial Use Reduction and Assessment of Particular Interventions

**Figure 1:**
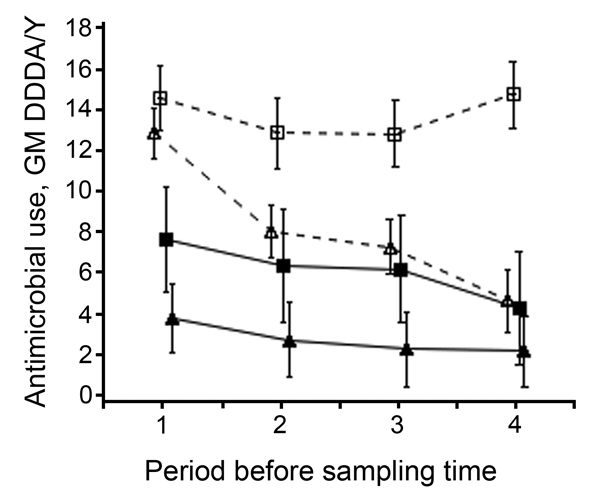
Antimicrobial use by type of farm during the 4 periods (≈6 months) before each sampling time in a study of the dose-response relationship between antimicrobial drug use and livestock-associated methicillin-resistant *Staphylococcus aureus* on pig farms, the Netherlands, 2011–2013. GM and 95% CI from log_2_ DDDA/Y. Farms were defined as open when they received external supplies of gilts ≥1 time per year from at least 1 supplier and as closed when they received no external supply of gilts. Closed triangles indicate closed farrow-to-finish farms; closed squares indicate closed farrowing farms; open triangles indicate open farrow-to-finish farms; open squares indicate open farrowing farms. DDDA/Y, defined daily dosages animal per year; GM, geometric mean. Error bars indicate 95% CIs.

During the 4 periods, tetracyclines were the most used antimicrobial drugs (37.6% of total DDDA/Y), followed by penicillins (30.2%), trimethoprim/sulfonamides (12.3%), macrolides/lincosamides (12.0%), and polymyxins (4.6%). The remaining 3.3% corresponded mainly with cephalosporins, amphenicols, pleuromutilines, and fluoroquinolones. Most antimicrobial classes decreased in parallel during the study; only macrolides slightly increased in DDDA/Y (9.9% to 16.5% from the first to the fourth period), and tetracyclines and trimethoprim/sulfonamides decreased slightly (from 37.0% to 32.7% and from 14.9% to 11.2%, respectively). Overall, 86% of the DDDA/Y were administered as batch or group treatment (i.e., animals were treated in groups mainly orally for prophylactic or metaphylactic reasons) and 14% as individual treatment (mainly by injection). These percentages did not significantly differ by type of farm. During the study, overall DDDA/Y decreased 44%, comparable with the national trend, across all farm types except open farrowing farms (Figure 1). Open and/or farrowing farms used at least twice as many antimicrobial drugs as closed and farrow-to-finish farms (Figure 1).

Farm management changes over time captured from the questionnaires were modest; just 10% of the intervention variables (median 9.7%, interquartile range [IQR] 6.0%–12.3%) per farm changed during the study. Thus, 27 farms had <12 of the 134 variables that changed. The median number of farms within a single change was 3 (IQR 1–4). Thus, 75% of the changes occurred in <4 farms. Changes over time did not differ by different farm type. Because of these limited and heterogeneous changes, an intervention effect could not be evaluated and we performed only a risk factor analysis.

### MRSA in Pigs

The number of MRSA-positive farms decreased slightly during the study (from 31 to 29 positive farms). Twenty-eight farms were MRSA-positive at all sampling times. Most were open (21 farms; 13 farrow-to-finish and 8 farrowing farms), and 7 were closed (5 farrow-to-finish and 2 farrowing). Four closed farrow-to-finish farms remained MRSA-negative during the entire study. From the remaining 4 farms, 3 became negative and 1 became positive during the study.

**Figure 2:**
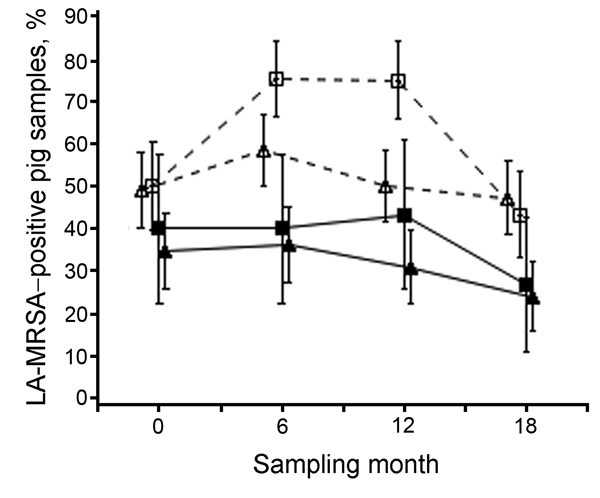
Prevalence of LA-MRSA–positive pooled samples from pigs during a study of the dose-response relationship between antimicrobial drug use and LA-MRSA on pig farms, the Netherlands, 2011–2013. Farms were defined as open when they received external supplies of gilts ≥1 time per year from at least 1 supplier and as closed when they received no external supply of gilts. Closed triangles indicate closed farrow-to-finish farms; closed squares indicate closed farrowing farms; open triangles indicate open farrow-to-finish farms; open squares indicate open farrowing farms. LA-MRSA, livestock-associated methicillin-resistant *Staphylococcus aureus*. Error bars indicate 95% Cis.

Overall pool-prevalence per sampling time decreased slightly on all farms. Open and farrowing farms remained at higher prevalences than closed and farrow-to-finish farms (Figure 2).

MRSA carriage differed notably between different age groups. The average pool-prevalence was 45.6% for finishing pigs; it was highest for suckling and weaned piglets (52.2% and 66.2%, respectively) and lowest for gilts and sows (30.2% and 30.8%, respectively). These prevalences did not significantly differ by farm type.

### MRSA in Humans

**Figure 3:**
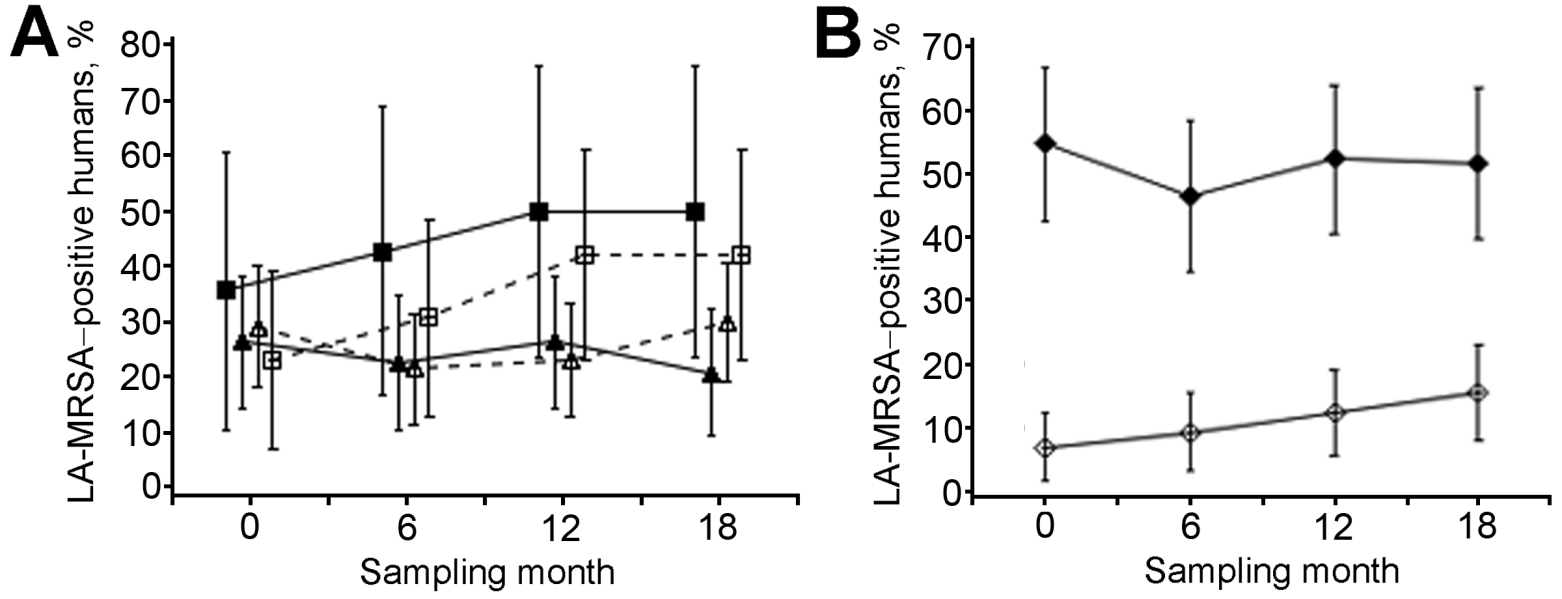
Prevalence of LA-MRSA in humans (n = 158) during a study of the dose-response relationship between antimicrobial drug use and LA-MRSA on pig farms, the Netherlands, 2011–2013. Results are stratified by type of farm (A) and number of hours worked on the farm (B). Farms were defined as open when they received external supplies of gilts ≥1 time per year from at least 1 supplier and as closed when they received no external supply of gilts. Closed triangles indicate closed farrow-to-finish farms; closed squares indicate closed farrowing farms; open triangles indicate open farrow-to-finish farms; open squares indicate open farrowing farms; open diamonds indicate persons working <20 hours per week; closed diamonds indicate persons working >20 hours per week. LA-MRSA, livestock-associated methicillin-resistant *Staphylococcus aureus*. Error bars indicate 95% Cis.

MRSA prevalence in humans did not change significantly over time (Figure 3, panels A, B). Prevalence and carriage dynamics differed by number of hours worked on the farm. Prevalence for persons working >20 hours per week was 5 times higher than for persons working <20 hours (Figure 3, panel B). Persons working >20 hours more frequently tested positive for MRSA at all sampling times (25%) or at least at 1 sampling time (48%), compared with those working <20 hours (2% and 24%, respectively). MRSA carriage dynamics did not significantly differ by level of antimicrobial use (data not shown) or by farm type (see overlap of 95% CIs in Figure 3, panel A).

### Antimicrobial Use and MRSA Carriage in Pigs and Humans

**Figure 4:**
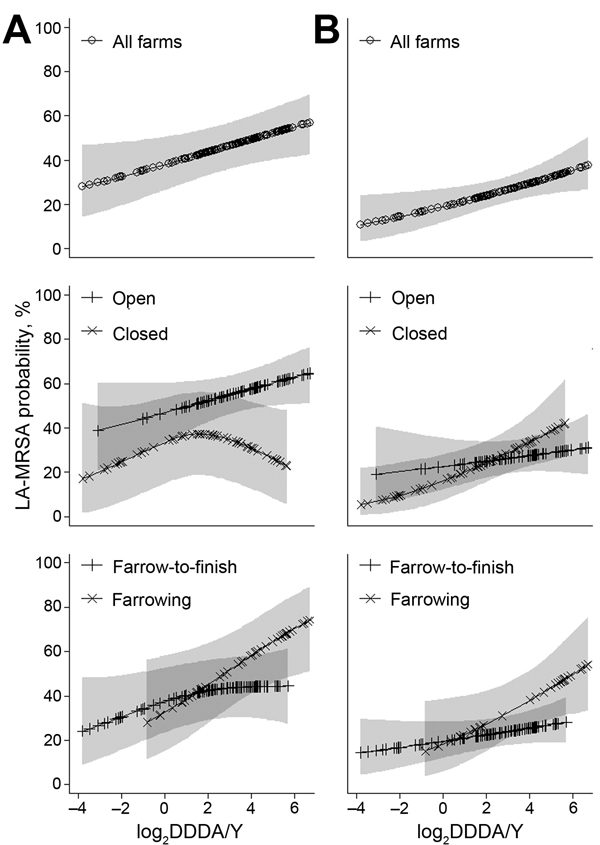
Dose–response relationships between antimicrobial use (log_2_ DDDA/Y) and livestock-associated methicillin-resistant *Staphylococcus aureus* (LA-MRSA) predicted probabilities in pigs (A) and humans (B), the Netherlands, 2011–2013. Splines were obtained from generalized additive mixed models with random intercepts for farms in the analysis for pigs and humans. Models accounted for the repeated measurements design and were adjusted for age group of pigs and for animal contact (i.e., hours worked) for humans. DDDA/Y was determined by dividing the total number of kilograms treatable with a single mass unit of the antimicrobial drug concerned, in accordance with the package insert information, by the average number of animal kilograms on the farm. Farms were defined as open when they received external supplies of gilts ≥1 time per year from at least 1 supplier and as closed when they received no external supply of gilts. p values and maximum-likelihood (ML) scores for the splines in the models for pigs: all farms (p = 0.03; ML 1433.5); open farms (p = 0.09; ML 991.3); closed farms (p = 0.09; ML 407.9); farrowing farms (p = 0.02; ML 438.5); farrow-to-finish farms (p = 0.39; ML 936.5). p values and ML scores for the splines in the models for humans: all farms (p = 0.01; ML 573.9); open farms (p = 0.41; ML 337.8); closed farms (p = 0.01; ML 229.9); farrowing farms (p = 0.03; ML 170.3); farrow-to-finish farms (p = 0.17; ML 398.2). DDDA/Y, defined daily dosages per animal per year; ML, maximum likelihood. Shaded areas indicate 95% CIs.

Farms with higher antimicrobial use were more likely to have MRSA-positive pigs (Figure 4). The odds that a pool would be MRSA positive was 16% higher for a 2-fold increase in DDDA/Y (Table 3). MRSA in pigs from open and from farrowing farms (high users of antimicrobial drugs) showed a positive trend and a significant association, respectively, with antimicrobial use (Table 3). The odds for testing LA-MRSA positive was higher when the proportion of group treatments with antimicrobial drugs was >0.5 (odds ratio [OR] 1.79, 95% CI 1.12–2.88; p = 0.02). This association was also found on open and on farrow-to-finish farms but was stronger in farrowing farms (OR 2.9, 95% CI 0.98–8.60; p = 0.05). Changes in MRSA carriage in pigs over time were significantly associated with changes in antimicrobial use; the odds for a 2-fold increase in antimicrobial use per sampling time (antimicrobial use–time interaction) decreased from the second to the last sampling (ORs 0.94, 1.27, 1.26, and 1.14 in the 4 consecutive samplings; p = 0.01). The same was found in an analysis restricted to open farms (ORs 0.86, 1.33, 1.18, and 1.06; p = 0.01). In farrowing farms (with little reduction in antimicrobial use), the antimicrobial use–time interaction was also significant, but ORs increased over time (ORs 1.04, 1.38, 1.62, 1.62; p = 0.03).

**Table 3:** ORs for livestock-associated MRSA in pigs and in humans with increasing use of antimicrobial drugs, the Netherlands, 2011–2013*

Characteristic	ORs for a 2-fold increase in DDDA/Y								
	Pooled pig samples					Farmers and family members			
	No.†	OR‡ (95% CI)	p value	−2 log RSPL§		No.¶	OR# (95%CI)	p value	−2 log RSPL§
All farms	1,421	1.16 (1.02–1.33)	0.03**	6,937.5		626	1.22 (1.01–1.48)	**0.04**	3,196.9
Supply of gilts††									
Open	867	1.11 (0.97–1.27)	0.12**	3,828.9		365	1.08 (0.85–1.38)	0.53	1,806.9
Closed	554	0.86 (0.69–1.33)	0.79	3,132.2		261	1.31 (0.94–1.81)	0.11	1,424.3
Production type									
Farrowing	476	1.38 (1.03–1.86)	0.03**	2,399.2		158	1.28 (0.85–1.94)	0.24	784.3
Farrow-to-finish	954	1.11 (0.95–1.30)	0.18	4,621.4		468	1.19 (0.95–1.50)	0.13	2,439.8

*Farm antimicrobial use was defined as 1 unit increase in the log_2_ DDDA/Y. Results from the random intercept generalized linear mixed models accounting for the repeated measurements design and adjusting for confounders. DDDA/Y indicates the number of days of antimicrobial use per year for an average animal on the farm. It was determined by dividing the total number of kilograms treatable with a single mass unit of the antimicrobial drug concerned, according to the package insert information, by the average number of animal kilograms on the farm. The denominator comprised sows and fatteners. DDDA/Y, defined daily dosages animal per year; MRSA, methicillin-resistant *Staphylococcus aureus*; OR, odd ratio; RSPL, residual pseudo-likelihood. Bold type indicates significance (p<0.05). †Number of observations at all sampling times together (10 pooled pig samples per farm on 36 farms in 4 sampling times). Values are missing for 19 observations. ‡For analysis in pigs, a farm random intercept was included in the mixed models and adjustment of ORs was made for sampling time and age group of pigs in the pool. §RSPL from the generalized linear mixed models. Models per stratum of external supply or type of production are not nested and -2 log RSPL cannot be used for comparison. ¶Number of observations in all sampling times together (158 persons, 4 sampling times). Values are missing for 6 observations. #For analysis in humans, a farm and a person random intercept were included in the mixed models, and number of hours worked on the farm and sampling time were used for adjustment of ORs. **These models additionally showed significant antimicrobial use–time interaction indicating parallel change in antimicrobial use and livestock-associated MRSA prevalence over the study period (see extended explanation in text). ††Farms were defined as open when they received external supplies of gilts ≥1 time per year from at least 1 supplier and as closed when they received no external supply of gilts.

We also observed a positive trend between antimicrobial use in animals and human MRSA carriage (Figure 4); the unadjusted OR for a 2-fold increase in DDDA/Y was 1.17 (95% CI 0.98–1.39; p = 0.09). The antimicrobial use–MRSA association did not significantly change after adjustment for hours worked (OR_adj_) (Table 3). When stratified by working hours, antimicrobial use remained especially associated with MRSA for persons working >20 hours per week (OR_adj_ 1.25, 95% CI 1.01–1.54; p = 0.04), compared with those working <20 hours (OR_adj_ 1.21, 95% CI 0.92–1.59; p = 0.18). A similar trend was observed across farrow-to-finish, farrowing, and closed farms (Table 3). The probability of LA-MRSA carriage was higher when the proportion of antimicrobial group treatments was ≥0.5 (OR_adj_ 1.76, 95% CI 0.79–3.90; p = 0.17). Reduction in antimicrobial use over time was not associated with any change in MRSA carriage in humans.

Specific levels of DDDA/Y for tetracyclines and penicillins were positively associated (p values from 0.06 to 0.23) with MRSA in pigs and humans (data not shown). The use of cephalosporins (on 7 farms, 6 of them open) during the first sampling time, was strongly associated with MRSA carriage in pigs (OR 2.94, 95% CI 1.45–5.87; p = 0.002). This association was not found for humans. Associations with other antimicrobial classes were weaker and often not statistically significant.

### Other Factors Determining MRSA in Humans and Pigs

Number of hours worked on the farm per week was strongly associated with MRSA in the human study population (univariate OR 1.82/10 hours worked increase, 95% CI 1.58–2.06; p<0.0001). Except for antimicrobial use, tasks related to animal contact and touching pigs from other farms were identified as risk factors for MRSA carriage in humans (Table 4). All variables in Table 4 were moderately or highly correlated (Spearman/Pearson ρ>0.5), and no multivariable model was built. We found no correlation between farm size, antimicrobial use, and hours worked.

**Table 4:** ORs for determinants of livestock-associated MRSA in humans, adjusted for number of hours worked per week on the farm, the Netherlands, 2011–2013*

Variable	No.†	OR‡ (95% CI)	p value§	−2 log RSPL¶
Age, per 10 y increase	632	1.14 (0.93–1.41)	0.2	3,204.1
MRSA prevalence in pigs, %, per 10% increase	632	1.08 (0.97–1.21)	0.16	3,190.9
MRSA-negative farm				
Yes	114	0.06 (0.01–0.27)	**<0.01**	3,288.1
No	518	Ref		
Touching dogs in past 6–12 mo				
Yes	446	0.51 (0.27–0.96)	**0.04**	3,173.7
No	180	Ref		
Touching pigs from other farms in past 6–12 mo				
Yes	86	2.82 (1.35–5.91)	**0.01**	3,205.3
No	546	Ref		
Sorting of sows in past 7 d				
Yes	221	1.91 (0.97–3.77)	0.06	3,144.5
No	392	Ref		
Sorting of suckling piglets in past 7 d				
Yes	159	2.21 (1.16–4.22)	**0.02**	3,169.5
No	455	Ref		
Sorting of weaned piglets in past 7 d				
Yes	174	1.63 (0.83–3.20)	0.16	3,162.9
No	439	Ref		
Feeding sows in past 7 d				
Yes	220	2.03 (0.99–4.17)	0.05	3,126.0
No	390	Ref		
Cleaning and disinfecting weaned piglets section in past 7 d				
Yes	81	1.70 (0.76–3.80)	0.2	3,157.8
No	538	Ref		

*Results from the random intercept generalized linear mixed models accounting for the repeated measurements design and adjusted for number of hours worked. MRSA, methicillin-associated *Staphylococcus aureus*; OR, odds ratio; Ref, reference category; RSPL, residual pseudo-likelihood. Bold type indicates p values <0.05. †Number of observations in all sampling times together (158 persons, 4 sampling times). Some variables have missing observations. ‡For analysis in humans, a farm and a person random intercept were included in the mixed models, and number of hours worked on the farm and sampling time were used for adjustment of ORs. §Only variables with p<0.2 in the mixed models are presented in the human analysis.  ¶RSPL from the generalized linear mixed models.

More biosecurity items reducing MRSA carriage in pigs were found on closed farms (e.g., different compartments per production phase, boarding platform for sows, washing overalls) (Table 5). Some variables had a similar effect on open and closed farms, increasing risk for MRSA (e.g., injection of antimicrobial drugs, clipping of teeth, and vaccination of piglets) or decreasing MRSA carriage (e.g., presence of a medication pipe separated from the water pipe, delivery room for materials, and keeping the sows in stable groups [i.e., not mixing]) (Table 5). However, other effects showed conflicting directions between strata (e.g., farm treatment plan, cleaning and disinfecting the carcass barrels, source of water supply) (Table 5). Low-level correlation existed between some variables (pairwise Spearman ρ<0.5) and with antimicrobial use or cephalosporin use (Table 5). A full multivariable model (Technical Appendix Table 3) was fitted by using the significant determinants from Table 5 together with the use of antimicrobials and cephalosporins; results from the backward elimination of non-significant terms are presented in Table 6. The presence of external supply of animals, overall antimicrobial use, and use of cephalosporins were significant risk factors retained through all elimination steps.

**Table 5:** ORs for determinants of livestock-associated MRSA positivity in pooled samples from pigs, the Netherlands, 2011–2013*

Characteristic	All farms			Open farms			Closed farms	
	No.†	OR (95% CI)		No.†	OR (95% CI)		No.†	OR (95% CI)
Farm								
No. sows, 300 increase§	1,421	1.4 (0.7–2.7)		867	1.3 (0.8–2.2)		554	2.6 (0.7–9.7)
External supply of gilts								
Open	867	**6.6 (2.3–19.0)**¶		867	Not computable		0	Not computable
Closed	554	Ref		0			554	
Type of production								
Farrow-to-finish	945	0.4 (0.1–1.6)		511	0.7 (0.3–1.6)		434	0.4 (0.0–17.7)
Farrowing	476	Ref		356	Ref		120	Ref
Farm treatment plan								
Yes	1,157	0.7 (0.4–1.3)		723	**0.6 (0.3–1.1)**		434	2.1 (0.6–7.1)
No	190	Ref		110	Ref		80	Ref
Water supply for animals								
Public, from tap	452	**2.8 (1.3–6.0)**#		218	0.8 (0.4–1.8)		234	**7.7 (2.5–24)**#
Private from private source	929	Ref		619	Ref		310	Ref
Separate medication pipe								
Yes	920	**0.4 (0.2–0.7)**#		526	**0.4 (0.2–0.7)**#		394	0.8 (0.2–3.7)
No	441	–		311	Ref		130	Ref
Biosecurity								
Different compartments per production phase								
Yes	880	0.9 (0.5–1.6)		600	1.7 (0.8–3.8)		280	**0.4 (0.2–1.1)**
No	521	Ref		257	Ref		264	Ref
Boarding platform for sows								
Yes	512	0.7 (0.4–1.4)		358	1.3 (0.7–2.6)		154	**0.2 (0.1–1.0)**
No	909	Ref		509	Ref		400	Ref
Clearly defined border of boarding platform								
Yes	989	0.7 (0.4–1.3)		569	1.1 (0.6–1.9)		420	**0.2 (0.1–0.6)**#
No	432	Ref		298	Ref		134	Ref
Carcass barrels cleaned and disinfected after emptied								
Yes	527	0.5 (0.3–1.0)**		317	**0.4 (0.2–0.8)**#		210	1.6 (0.5–5.1)
No	864	Ref		530	Ref		334	Ref
Delivery room for materials								
Yes	1,031	**0.4 (0.2–0.7**)**		677	0.5 (0.2–1.0)**		354	**0.3 (0.1–0.6)**#
No	320	Ref		140	Ref		180	Ref
Pigs go outside when moved								
Yes	627	0.8 (0.4–1.6)		367	1.4 (0.8–2.6)		274	0.2 (0.1–0.8)**
No	744	Ref		470	Ref		260	Ref
Workers’ overalls washed								
Yes	687	0.8 (0.5–1.4)		317	1.2 (0.7–2.1)		370	**0.3 (0.1–1.2)**
No	734	Ref		550	Ref		184	Ref
Removal of manure in winter								
Manure stays <6 mo	1,007	1.2 (0.7–2.0)		647	0.8 (0.5–1.5)		360	**2.9 (1.0–8.9)**
Manure stays >6 mo	380	Ref		186	Ref		194	Ref
Animal management and contact structure								
Injection of piglets with antimicrobial drugs during the first week.								
Yes	830	**2.0 (1.2–3.3)**#		610	1.4 (0.8–2.5)		220	**3.7 (1.6–8.6)**#
No	571	Ref		257	Ref		314	Ref
Tooth clipping in piglets								
Yes	516	3.2 (1.4–7.0)**		34,650	**3.0 (1.5–6.2)**#		170	4.0 (0.5–30.6)
No	875	Ref		1	Ref		374	Ref
Vaccination of piglets and/or fatteners								
Yes	1,090	2.5 (1.4–4.5)**		690	2.0 (1.1–3.4)**		400	7.2 (1.6–32)**
No	311	Ref		167	Ref		144	Ref
Needles for vaccination renewed per compartment								
Yes	848	1.9 (1.2–3.1)**		508	1.7 (1.0–2.7)**		340	2.1 (0.4–12.1)
No	456	Ref		312	Ref		144	Ref
Some piglets reared motherless								
Yes	385	1.3 (0.7–2.3)		311	**1.6 (0.9–2.7)**		74	0.2 (0.0–0.9)**
No	1,026	Ref		546	Ref		480	Ref
Sows in stable groups								
Yes	772	**0.5 (0.3–0.8)**#		432	**0.6 (0.3–1.0)**		340	**0.5 (0.2–1.1)**
No	619	Ref		405	Ref		214	Ref
Hygiene								
In the piglet section								
Disinfectant	189	**0.3 (0.2–0.7)**#		139	**0.3 (0.1–0.7)**#		50	0.9 (0.1–5.9)
Soaking agent	280	**2.0 (1.0–4.4)**		180	3.1 (1.3–7.5)**		100	0.1 (0.0–0.8)**
Disinfectant + soaking agent	698	1.2 (0.6–2.3)		408	1.4 (0.6–3.1)		290	0.8 (0.3–2.5)
None	254	Ref		140	Ref		114	Ref
In the mating section								
Disinfectant + soaking agent	239	**0.6 (0.3–1.1)**		89	0.3 (0.1–0.8)**		150	2.2 (0.6–7.7)
None	1,182	Ref		778	Ref		404	Ref
In the gilt section								
Soaking agent	220	1.0 (0.5–2.0)		100	1.5 (0.7–3.6)		120	**0.3 (0.1–1.1)**
Disinfectant + soaking agent	585	1.0 (0.6–1.6)		335	1.0 (0.6–1.7)		250	1.3 (0.5–3.9)
None	616	Ref		432	Ref		184	Ref

*Fits for the univariate adjusted models in all farms: −2 log RSPL estimations ranged from a minimum of 6386.56 to a maximum of 7016.07. Results from the longitudinal analysis with generalized linear mixed models taking into account the repeated measurements design and adjusted for age group of the pool. Variables with p<0.1 in the overall analysis or in at least 1 stratum (open or closed) are presented. OR and p values are in bold type when p<0.1. Farms were defined as open when they received external supplies of gilts ≥1 time per year from at least 1 supplier and as closed when they received no external supply of gilts. MRSA, methicillin-resistant *Staphylococcus aureus*; OR, odds ratio; Ref, reference category; RSPL, residual pseudo-likelihood. †Number of observations at all sampling times together (10 pooled pig samples per farm in 36 farms in 4 sampling times). Some variables have missing observations. §Items evaluated irrespective of significance. ¶p<0.001. #p<0.01. **p<0.05.

**Table 6:** ORs for the most important determinants of livestock-associated MRSA positivity in 1,054 pooled pig samples from 32 farms (multivariable final model), the Netherlands, 2011–2013*

Characteristic	No.†	OR (95% CI)	p value
Sampling time			
0 mo	262	0.83 (0.48–1.43)	<0.001
6 mo	290	2.05 (1.25–3.37)	
12 mo	259	1.96 (1.20–3.20)	
18 mo	243	Ref	
Age group			
Gilts	212	1.08 (0.65–1.80)	<0.001
Finishers	140	4.09 (2.30–7.25)	
Suckling piglets	212	3.87 (2.34–6.39)	
Weaned piglets	280	9.89 (5.96–16.39)	
Sows	210	Ref	
External supply of gilts‡			
Open	630	5.54 (1.56–19.27)	0.008
Closed	424	Ref	
Delivery room for materials			
Yes	804	0.29 (0.13–0.62)	0.001
No	250	Ref	
Sows housed in stable groups			
Yes	594	0.53 (0.29–0.96)	0.038
No	460	Ref	
Antimicrobial drug use, per 2-fold increase, log_2_ DDDA/Y	1,054	1.22 (1.03–1.44)	0.024
Use of cephalosporins			
Yes	84	3.15 (1.47–6.74)	0.003
No	970	Ref	

*Model fit: −2 log RSPL estimation = 5331.7. Multivariable final model after backward elimination of non-significant variables from a full model (online Technical Appendix Table 3, http://wwwnc.cdc.gov/EID/article/21/6/14-0706-Techapp1.pdf) containing the significant associations (p<0.05) presented in Table 5 (http://wwwnc.cdc.gov/EID/article/21/6/14-0706-T5.htm) for all farms, together with antimicrobial drug use, use of cephalosporins, sampling time, and age group of the pool. MRSA, methicillin-resistant *Staphylococcus aureus*; OR, odds ratio; DDDA/Y, defined daily dosages animal per year; Ref, reference category; RSPL, residual pseudo-likelihood. †Multiple variables had missing values in the full model reducing the number of observations in the final model. ‡Farms were defined as open when they received external supplies of gilts ≥1 time per year from at least 1 supplier and as closed when they received no external supply of gilts.

## Discussion

We found a quantitative association between antimicrobial use and MRSA in pigs and humans living and/or working on pig farms. Our findings indicate that a reduction in antimicrobial use is likely to be effective in reducing MRSA carriage in pigs. Risk for MRSA is higher for increased use of tetracyclines and penicillins but more so for use of cephalosporins. Except for the change in antimicrobial use over time, overall changes in farm management were modest and not sufficient to contribute to decreasing MRSA levels. Nevertheless, several factors were identified as possible candidates for future intervention studies.

The extent of representativeness of a convenient sample is difficult to evaluate. Nonetheless, descriptive results show the heterogeneity of farms included; the decreasing trend in use of antimicrobial drugs and the proportions by antimicrobial classes and by group and individual treatments mirror national data (*16*,*17*).

Levels of antimicrobial use differed considerably by farm type. Open and/or farrowing farms were high users of antimicrobial drugs and showed a strong positive dose–response relationship between antimicrobial use and MRSA in pigs. In particular, the use of cephalosporins was related to higher carriage rates of MRSA. The literature shows that selective pressure favors transmission and spread of MRSA in pigs (*13*,*21*). MRSA ST398 isolates have shown high diversity of resistance genes, and all of them are resistant to penicillin and tetracycline (*22*); the DDDA/Y of these antimicrobial classes was related to MRSA in our results. Although the use of cephalosporins represented a small proportion of total antimicrobial use, it was strongly associated with MRSA in pigs. These antimicrobial drugs are known to be important for generation and propagation of resistance in *S. aureus* and other microorganisms (*23*). The fact that they were administered before the first sampling time might be related to the initial increase in MRSA prevalence in pigs. We refrained from presenting detailed associations by antimicrobial classes because mostly all classes were used on all the farms and were correlated; thus, effects of individual classes of antimicrobial drugs were difficult to disentangle and require cautious interpretation. The higher risk posed by administering group treatments confirms previous findings in the literature (*4*,*12*). Interaction between antimicrobial use and time was significant, suggesting a decrease of MRSA prevalence in pigs over time with decreasing antimicrobial use. These associations were not found on closed and farrow-to-finishing farms, indicating that below a certain level, antimicrobial use contributes less to MRSA prevalence. Nevertheless, it is important to consider that other studies have reported high MRSA transmission in the absence of antimicrobial agents (*24*,*25*). Thus, antimicrobial use should not be the only target for intervention.

Direct contact with positive animals has been widely reported as the major force driving MRSA carriage in persons living and/or working on farms (*26*–*28*). In our study, higher risk for MRSA in the human study population was strongly associated with the number of hours worked on the farm and to the variables related to tasks performed on the farm. However, antimicrobial use also showed a significant positive dose–response relationship to MRSA human carriage during the study, even after adjustment for hours worked. When antimicrobial drugs are administered to animals, substantial quantities of these drugs remain in manure, on surfaces of barns, and in dust as a potential risk source (*29*). The selective pressure exerted by exposure to dust containing antimicrobial drugs or directly to antimicrobial powder formulations would explain the higher risk for MRSA carriage in persons living or working on pig farms. However, this independent effect of antimicrobial use on susceptible bacteria in humans is difficult to disentangle from direct MRSA transmission from animals to humans.

The role of animal trade in introducing and spreading MRSA has been reported (*4*,*10*–*13*), but information about carriage status of animals entering the farm was not available in this study. Nevertheless, our results corroborate that external supply of animals is significantly associated with higher MRSA levels. A higher selective pressure for MRSA might also occur on open farms because they had higher overall antimicrobial use and 6 of them used cephalosporins. However, external supply of animals appeared to be a risk factor, even when evaluated together with antimicrobial use and cephalosporin use in the multivariate model.

A previous study in the Netherlands found that the prevalence of MRSA-positive pig farms steeply increased from 40% in 2007 to 70% in 2008 (*4*). Our results show that this prevalence remains high (>80%) but the slight increase since 2008 indicates that MRSA carriage in pigs might have reached a steady state. Herd size was identified as a risk factor when MRSA was emerging in livestock (*12*); however, we found no such association.

Several determinants could be targeted for specific interventions in the near future. Factors regarding biosecurity considerably reduced the risk for MRSA, especially on closed farms. It is remarkable that mostly variables related to management of piglets were associated with MRSA. Piglets are more susceptible to infection, and they receive larger amounts of antimicrobial drugs. Tooth clipping in piglets increased the probability for MRSA carriage; MRSA transmission from piglet to piglet might be higher when the same plier is used or through the worker. Unexpected risk factors could be the product of reverse causality such as vaccination of piglets, fatteners, or both and frequent change of needles. These possibilities need to be explored in other, independent studies. Observations for cleaning and disinfection were not consistent. It has been previously reported that disinfection has a short-lasting positive effect for MRSA reduction (*30*). Keeping the groups of sows stable was an interesting protective factor that might reduce MRSA spread within the farm. Animals that drank water from the public supply instead of from a private source had increased probability for MRSA. Zinc oxide specifically co-selects for MRSA ST398 (*31*,*32*), and concentrations can be higher in tap water as a result of leaching from pipes. A higher zinc intake in animals might have led to higher selection for MRSA, but this association needs further research.

Pooling of animal samples leads to less precise prevalence estimates (*33*,*34*) but is a low-cost alternative for individual sampling that enabled enlargement of the number of farms tested. Individual testing, however, would not be expected to lead to different outcomes.

This study shows the inherent difficulty in evaluating pragmatic interventions for MRSA control in pig farms under field conditions over a relatively short period. More farms and controlled interventions, together with longer follow-up periods to capture prevalence changes, are needed to assess intervention effects over time. Despite the limitations, we identified factors that can define attainable future interventions (e.g., avoiding tooth clipping, keeping sows in stable groups). Finally, we demonstrated that antimicrobial use has a strong and positive dose–response relationship with MRSA in pigs and humans living and/or working on pig farms. In particular, use of cephalosporins resulted in increased MRSA carriage rates in pigs. Animal and public health authorities should continue to promote the reduction of antimicrobial use. Different approaches for MRSA control might be needed in light of the differences by type of production and external supply of animals.

**Technical Appendix.** Farm and human questionnaires used at each of the 4 sampling times and full multivariable model for positivity of pooled samples from pigs. Risk factor analysis and assessment of the dose-response relationship between antimicrobial drugs and livestock-associated methicillin-resistant Staphylococcus aureus, the Netherlands, 2011-2013.
