# Supplementary material for: Dose-Response Relationship between Antimicrobial Drugs and Livestock-Associated MRSA in Pig Farming
Source: Emerg Infect Dis. 2015 Jun;21(6):950–9. doi: 10.3201/eid2106.140706 (PMC4451891; doi:10.3201/eid2106.140706)
Supplement: Supplementary file 1 — Technical Appendix. Farm and human questionnaires used at each of the 4 sampling times and full multivariable model for positivity of pooled samples from pigs. Risk factor analysis and assessment of the dose-response relationship between antimicrobial drugs and livestock-associated methicillin-resistant Staphylococcus aureus, the Netherlands, 2011-2013. [file 14-0706-Techapp-s1.pdf]

Article DOI: http://dx.doi.org/10.3201/eid2106.140706

Dose-Response Relationship between Antimicrobial Drugs and Livestock-Associated MRSA in Pig Farming

Technical Appendix

Technical Appendix Table 1. Farm questionnaire used in each of the four sampling times in a risk factor analysis for livestock-associated methicillin-resistant *Staphylococcus aureus* in pig farming, the Netherlands, 2011-2013.

Question	Possible answers
General farm characteristics:	
**1. Farm size: mean number of sows present per year ¶†**	No.
2. **Type of production¶**	Farrowing / Farrow-to-finish
3. Mean number of fattener pigs present per year	No.
4. Frequency of gilts supply per year	No.
5. Number of farms from which pigs are supplied per year	No.
**6. External supply of gilts ≥1 time a year from at least one supplier (aggregated from questions 4 and 5) ¶**	Open / Closed
7. Complete all in-all out system is applied for closed farm¶	Yes / No
8. Frequency of removal of piglets	No.
9. Frequency of removal of fattener pigs	No.
10. Frequency of removal of rearing gilts	No.
11. Frequency of removal of sows	No.
12. Percentage of loss of weaned piglets per year	No.
13. Average lactation period (days)	No.
14. Mean number of weaned piglets per sow per year	No.
15. Mean number of weaned piglets per litter	No.
16. Percentage of loss of fattener pigs per year	No.
17. Percentage of loss of sows per year	No.
18. Mean growth per piglet per day	No.
19. Mean growth per fattener pig per year	No.
20. After delivering the leftover piglets are placed together	Yes / No
21. After delivering there is a leftover piglets department	Yes / No
Biosecurity and hygiene status	
22. Hygiene status of the farm	A/ B/ C/ D/ E/ F
23. The farm owns an Specific pathogen free (SPF) status¶	Yes / No
24. SPF status for	App/ Aujeszky/ M.Hyo/ PRRSv/ None
25. Housing of the gestating sows ¶	Cubicle / Groups
26. Group size of the gestating sows¶	Yes / No
27. There are other animals present on the farm¶	Yes / No
28. There are also sheep present on the farm¶	Yes / No
29. There are also goats present on the farm¶	Yes / No
30. There are also cattle present on the farm¶	Yes / No
31. There are also horses present on the farm¶	Yes / No
32. There are also poultry present on the farm¶	Yes / No
33. No other farming animals are present on the farm	Yes / No
34. Cats are able to enter the shed	Yes / No
35. Dogs are able to enter the shed¶	Yes / No
36. Number of people working on the farm (including assisting family members)	Yes / No
37. Biosecurity score (aggregated sum from questions 38, 42, 45,49, 90, 136 where yes=1 and no=0)¶	0 to 6
38. There is only one entrance to the farm, which is the hygiene lock, other doors are locked¶	Yes / No
39. A doorbell or phone number of the owner is clearly visible at the entrance of the farm. In this way it is possible to contact the people of the farm¶	Yes / No
40. The farm's terrain is paved and cleaned up ¶	Yes / No
41. Silos are filled from the side of the dirty road¶	Yes / No
42. Pigs and personnel go outside during working activities¶	Yes / No
43. The hygiene lock consists of a clean and dirty part, separated by a corridor shower¶	Yes / No
44. The lock does not contain a shower, but it does consist of a clearly separated clean and dirty part	Yes / No
45. Showering is mandatory¶	Yes / No
46. If showering is not mandatory, everyone washes his or her hands before entering the farm	Yes / No

Question	Possible answers
47. Showering is not mandatory, however wearing farm-issued clothing is (pants and shirt	Yes / No
48. Farmer and his co-workers use the hygiene lock in the same way visitors do¶	Yes / No
49. The farmer and his co-workers wash their hands before entering the farm¶	Yes / No
50. There is warm water available¶	Yes / No
51. There is soap available	Yes / No
52. There is a clean towel present	Yes / No
53. There are clean boots and overalls available¶	Yes / No
**54. Workers' overalls are washed daily¶**	Yes / No
55. Overalls are washed	Daily/Weekly / Monthly/ Less than monthly
56. On average, for how many months does the manure stay in the pits during the summer?¶	<6 / ≥6
57. On average, for how many months does the manure stay in the pits during the summer?¶	<3 / 3-6 / 6-9 / >9
**58. On average, for how many months does the manure stay in the pits during the winter?¶**	<6 / ≥6
59. On average, for how many months does the manure stay in the pits during the winter?¶	<3 / 3-6 / 6-9 / >9
60. Delivered animals are placed in quarantine for a certain period of time. This part has its own entrance and is not a part of the rest of the farm	Yes / No
61. The quarantine has its own lock and clothing	Yes / No
62. The quarantine is visited at the end of the day	Yes / No
63. After delivery, the gilts arrive at an empty and cleaned section. This is not a quarantine	Yes / No
64. When gilts are delivered, these animals do not arrive at an empty section or quarantine	Yes / No
65. Piglets are delivered on the same day as fatteners	Yes / No
66. Sperm is delivered on the dirty road, the cooling box is not brought on the farm terrain¶	Yes / No
**67. There is a delivery room for materials and bagged goods. Materials are not delivered directly to the farm¶**	Yes / No
68. Pest control is handed over to a professional organization¶	Yes / No
69. Birds are able to enter the sheds¶	Yes / No
70. Is there presence of rats and/or mice?¶	Yes / No
71. Animals have access to an outdoor run (e.g. after weaning)	Yes / No
**72. When pigs are moved, they have to go outside¶**	Yes / No
**73. There is a boarding platform for the sows, preventing the truck from parking directly against the shed¶**	Yes / No
74. There is a boarding platform for the piglets, preventing the truck from parking directly against the shed¶	Yes / No
75. There is a boarding platform for the fatteners, preventing the truck from parking directly against the shed ¶	Yes / No
76. There is boarding platform for piglets and/or fatteners¶	Yes / No
77. The boarding location is not situated directly next to or beneath an air inlet¶	Yes / No
**78. The border for the delivery of animals is a 100% clear and is also implemented this way¶**	Yes / No
79. After delivery of the animals, the delivery platform is cleaned and disinfected immediately¶	Yes / No
80. The driver does not enter the clean road¶	Yes / No
81. Transport trucks are clean, empty and disinfected when they arrive on the farm to load the sows¶	Yes / No
82. The carcass storage is cooled and locked¶	Yes / No
83. The carcass cooler is situated on the dirty road¶	Yes / No
84. Small destruction materials can be thrown into the cooler from the clean road¶	Yes / No
85. There is a double number of barrels on the farm. So there is a surplus of barrels¶	Yes / No
**86. After the destructor emptied the barrels, the barrels are cleaned and disinfected before retrieved¶**	Yes / No
87. Rinsing water of cleaning barrels is discharged into the sewer	Yes / No
88. When handling carcasses, gloves are always worn¶	Yes / No
89. When treating sick animals, gloves are always worn¶	Yes / No
90. When treating piglets, gloves are always worn¶	Yes / No
91. After someone entered a pen of the weaned piglets or the fatteners, hygienic measures are taken	Yes / No
<u>Animal health management</u>	
92. During gestation, vaccinations are implemented	Yes / No
93. During lactation, vaccinations are implemented	Yes / No
**94. The piglets and/or fatteners are vaccinated¶**	Yes / No
95. PRRSv vaccination is implemented	Yes / No
96. *Mycoplasma hyponeumoniae* vaccination is implemented	Yes / No
97. PCV2 vaccination is implemented	Yes / No
98. APP vaccination is implemented	Yes / No
99. Glässer vaccination is implemented	Yes / No
100. The piglets are vaccinated without the use of a needle	Yes / No
**101. The teeth of the new-born piglets are clipped¶**	Yes / No
102. The tails of the piglets are docked¶	Yes / No
103. The boar piglets are castrated	Yes / No
**104. All piglets are given an injection of antibiotics in their first week of life¶**	Yes / No

Question	Possible answers
105. When treating the piglets, gloves are worn	Yes / No
106. The gloves are renewed:	After each litter/ After each section /Each day
107. When treating the piglets, other hygiene measures are taken in order to prevent the transfer of infection from one to the other litter¶	Yes / No
108. Needles for vaccination of sows are renewed:¶	Once a day / Once a week / When necessary
109. Needles for vaccination of piglets and/or fatteners are renewed per pen¶	Yes / No
**110. Needles for vaccination of piglets and/or fatteners are renewed per section¶**	Yes / No
111. At the end of the day, the syringes are cleaned:	Daily, rinsing with cold water / Taken apart and with water and soap / Dishwasher / Not cleaned
112. There is a sick-bay present¶	Yes / No
113. The sick-bay is used as a sick-bay	Yes / No
114. In the sick-bay, different ages are present	Yes / No
115. Animals enter and exit a sick-bay (back to the farm)	Yes / No
116. The sick-bay is visited at the end of the day	Yes / No
117. There is a care option for sick and cripple animals at their own group/section¶	Yes / No
<u>Animal contact structure</u>	
**118. The sows are housed in stable groups¶**	Yes / No
119. Piglets are placed per litter¶	Yes / No
**120. Some piglets are reared motherless¶**	Yes / No
121. After the third day, piglets can still be switched¶	Yes / No
122. Foster sows are used¶	Yes / No
123. When creating foster sows, different litters of piglets are moved up to a different sow	Yes / No
124. Separation between piglet cages is open¶	Yes / No
125. Separation between piglet cages is taken up by the feeder and/or trough, which is shared between the animals¶	Yes / No
126. Supervision of the animals from the central hall way	Yes / No
127. Separation between cages fatteners is open¶	Yes / No
128. Separation between cages fatteners is taken up by the feeder and/or trough, which is shared between the animals¶	Yes / No
129. Separation between cages for sows is open¶	Yes / No
130. Separation between cages for sows is taken up by the feeder and/or trough, which is shared between the animals¶	Yes / No
131. Carcasses are placed on the ground in the section¶	Yes / No
132. Carcasses are placed on the ground in the central hall way¶	Yes / No
133. Cadaver bags are used¶	Yes / No
134. Considering hygienic measures, direction of work is from young to old ¶	Yes / No
**135. Sows, piglets and fatteners are different components within the farm. Each component makes use of different clothing and materials¶**	Yes / No
<u>Cleaning and disinfection</u>	
136. All farm sections are cleaned and disinfected ¶	Yes / No
137. All farm sections are disinfected ¶	Yes / No
138. All farm sections are cleaned with soaking agents¶	Yes / No
139. Farrow section hygiene (aggregated variable from questions 143 and 144)¶	Disinfection and or soaking / None
140. Farrow corridor hygiene (aggregated variable from questions 148 and 149)¶	Yes / No
141. Farrowing section is cleaned with cold water	Yes / No
142. Farrowing section is cleaned with warm water	Yes / No
143. Farrowing section is cleaned with soaking agent	Yes / No
144. Farrowing section is cleaned with disinfection agent	Yes / No
145. After cleaning farrowing section, there is a dry period of at least 24 hours	Yes / No
146. Farrowing section is cleaned by sweeping	Yes / No
147. Farrowing corridor is cleaned with soaking agent	Yes / No
148. Farrowing corridor is cleaned with disinfection agent	Yes / No
**149. Piglets section hygiene (aggregated variable from questions 153 and 154)¶**	Disinfection and soaking / Just soaking / Just disinfection / None
150. Piglets corridor hygiene (aggregated variable from questions 157 and 158)¶	Disinfection and soaking / Just soaking / None
151. Piglets section is cleaned with cold water	Yes / No
152. Piglets section is cleaned with warm water	Yes / No
153. Piglets section is cleaned with soaking agent	Yes / No
154. Piglets section is cleaned with disinfection agent	Yes / No
155. After cleaning piglets section, there is a dry period of at least 24 hours	Yes / No
156. Piglets section is cleaned by sweeping	Yes / No
157. Piglets corridor is cleaned with soaking agent	Yes / No
158. Piglets corridor is cleaned with disinfection agent	Yes / No
159. Fatteners section hygiene (aggregated variable from questions 163 and 164)	Disinfection and soaking / Just soaking / Just disinfection / None
160. Fattener corridor hygiene (aggregated variable from questions 167 and 168)	Disinfection and or soaking /

Question	Possible answers
	None
161. Fattener section is cleaned with cold water	Yes / No
162. Fatteners section is cleaned with warm water	Yes / No
163. Fatteners section is cleaned with soaking agent	Yes / No
164. Fatteners section is cleaned with disinfection agent	Yes / No
165. After cleaning fatteners section, there is a dry period of at least 24 hours	Yes / No
166. Fatteners section is cleaned by sweeping	Yes / No
167. Fatteners corridor is cleaned with soaking agent	Yes / No
168. Fatteners corridor is cleaned with disinfection agent	Yes / No
**169. Gilts section hygiene (aggregated variable from questions 173 and 174)¶**	Disinfection and or soaking / Just soaking / None
170. Gilts corridor hygiene (aggregated variable from questions 177 and 178)¶	Disinfection and or soaking / None
171. Gilts section is cleaned with cold water	Yes / No
172. Gilts section is cleaned with warm water	Yes / No
173. Gilts section is cleaned with soaking agent	Yes / No
174. Gilts section is cleaned with disinfection agent	Yes / No
175. After cleaning gilts section, there is a dry period of at least 24 hours	Yes / No
176. Gilts section is cleaned by sweeping	Yes / No
177. Gilts corridor is cleaned with soaking agent	Yes / No
178. Gilts corridor is cleaned with disinfection agent	Yes / No
**179. Mating section hygiene (aggregated variable from questions 183 and 184) ¶**	Disinfection and or soaking / None
180. Mating corridor hygiene (aggregated variable from questions 187 and 188)¶	Disinfection and or soaking / None
181. Mating section is cleaned with cold water	Yes / No
182. Mating section is cleaned with warm water	Yes / No
183. Mating section is cleaned with soaking agent	Yes / No
184. Mating section is cleaned with disinfection agent	Yes / No
185. After cleaning mating section, there is a dry period of at least 24 hours	Yes / No
186. Mating section is cleaned by sweeping	Yes / No
187. Mating corridor is cleaned with soaking agent	Yes / No
188. Mating corridor is cleaned with disinfection agent	Yes / No
189. Gestation shed section hygiene (aggregated variable from questions 193 and 194)¶	Disinfection and or soaking / None
190. Gestation shed corridor hygiene (aggregated variable from questions 197 and 198)¶	Disinfection and or soaking / None
191. Gestation shed section is cleaned with cold water	Yes / No
192. Gestation shed section is cleaned with warm water	Yes / No
193. Gestation shed section is cleaned with soaking agent	Yes / No
194. Gestation shed section is cleaned with disinfection agent	Yes / No
195. After cleaning gestation shed section, there is a dry period of at least 24 hours	Yes / No
196. Gestation shed section is cleaned by sweeping	Yes / No
197. Gestation corridor is cleaned with soaking agent	Yes / No
198. Gestation corridor is cleaned with disinfection agent	Yes / No
Workflow, feed and water supply	
199. Work is visibly done with a week planner¶	Yes / No
200. Work is visibly done with a day planner¶	Yes / No
201. There are protocols present in the shed (work flows) ¶	Yes / No
202. The date of placement is present on the section doors ¶	Yes / No
**203. Farm treatment plan recorded and stored¶**	Yes / No
204. A medical prescription with dosage is present on the farm¶	Yes / No
205. Farrowing sows are fed with broth¶	Yes / No
206. Farrowing sows are fed with dry feed¶	Yes / No
207. Farrowing sows are fed with milk	Yes / No
208. Farrowing sows are fed with mush/pulp	Yes / No
209. Dry and gestating sows are fed with broth¶	Yes / No
210. Dry and gestating sows are fed with dry feed¶	Yes / No
211. Dry and gestating sows are fed with milk	Yes / No
212. Dry and gestating sows are fed with mush/pulp	Yes / No
213. Gilts are fed with broth¶	Yes / No
214. Gilts are fed with dry feed¶	Yes / No
215. Gilts are fed with milk	Yes / No
216. Gilts are fed with mush/pulp	Yes / No
217. Piglets with sow are fed with broth¶	Yes / No
218. Piglets with sow are fed with dry feed¶	Yes / No
219. Piglets with sow are fed with milk¶	Yes / No
220. Piglets with sow are fed with mush/pulp¶	Yes / No
221. Weaned piglets are fed with broth¶	Yes / No
222. Weaned piglets are fed with dry feed¶	Yes / No
223. Weaned piglets are fed with milk	Yes / No
224. Weaned piglets are fed with mush/pulp¶	Yes / No
225. Fatteners are fed with broth¶	Yes / No
226. Fatteners are fed with dry feed	Yes / No

Question	Possible answers
227. Fatteners are fed with milk	Yes / No
228. Fatteners are fed with mush/pulp	Yes / No
**229. Animals get water mainly from¶**	Public source, tap/ Private source
230. Water medication is possible via a dosator¶	Yes / No
231. Water medication is possible per section¶	Yes / No
**232. A separate medication pipe is present on the farm¶**	Yes / No
233. The water pipe is cleaned¶	Yes / No
234. In the farrowing section drinking water is just supplied via a nipple¶	Yes / No
235. In the farrowing section drinking water is mainly supplied via a nipple¶	Yes / No
236. In the farrowing section drinking water is mainly supplied via a water bowl¶	Yes / No
237. In the piglet section drinking water is just supplied via a nipple¶	Yes / No
238. In the piglet section drinking water is mainly supplied via a nipple¶	Yes / No
239. In the piglet section drinking water is just supplied via water bowl¶	Yes / No
240. In the piglet section drinking water is mainly supplied via a water bowl¶	Yes / No
241. In the fattener section drinking water is mainly supplied via a nipple¶	Yes / No
242. In the fattener section drinking water is just supplied via water bowl¶	Yes / No
243. In the fattener section drinking water is mainly supplied via a water bowl¶	Yes / No
244. In the (rearing) gilt section drinking water is mainly supplied via a nipple¶	Yes / No
245. In the (rearing) gilt section drinking water is mainly supplied via a water bowl¶	Yes / No
246. In the mating section drinking water is mainly supplied via a nipple¶	Yes / No
247. In the mating section drinking water is mainly supplied via a water bowl¶	Yes / No
248. In the gestation shed drinking water is mainly supplied via a water bowl¶	Yes / No

Bold type indicates variables presented in table 5 with p≤0.1 in the overall analysis or in at least one stratum (open or closed).
¶Variables with less than 10% missing values, at least 10% of farms in each category. Random intercept generalized linear mixed models were fitted for each variable by using a macro statement in SAS.
†Farm size had a p>0.10 but was evaluated and presented irrespective of significance.

Technical Appendix Table 2. Human questionnaire used in each of the four sampling times in a risk factor analysis for livestock-associated methicillin-resistant *Staphylococcus aureus* in pig farming, the Netherlands, 2011-2013.

Question	Possible answers
General characteristics:	
1. Sex: ¶	M / F
**2. Age¶**	Years
3. Relation to pig farmer¶	Farmer / Worker / Partner/ Children
4. I live on the pig farm ¶	Yes / No
5. Number of household members: ¶	No.
Animal contact	
6. Average number of hours worked on the pig farm: ¶	No.
**7. Did you touch someone else's pigs in the last 6/12 months? ¶**	Yes / No
8. Did you touch dairy cattle in the last 6/12 months? ¶	Yes / No
9. Did you touch any calves in the last 6/12 months? ¶	Yes / No
10. Did you touch any horses in the last 6/12 months?	Yes / No
11. Did you touch any sheep in the last 6/12 months? ¶	Yes / No
12. Did you touch any goats in the last 6/12 months? ¶	Yes / No
**13. Did you touch any dogs in the last 6/12 months? ¶**	Yes / No
14. Did you touch any cats in the last 6/12 months? ¶	Yes / No
15. Did you touch any poultry in the last 6/12 months?	Yes / No
16. Do you have pets? ¶	Yes / No
a. How many dogs? ¶	No.
b. How many cats? ¶	No.
c. How many rabbits?	No.
d. How many rodents?	No.
e. How many fishes?	No.
17. Are your pets allowed in the living room? ¶	Yes / No
Tasks performed in the farm:	
**18. Sorting the sows in the past 7 days ¶**	Yes / No
**19. Sorting the suckling piglets in the past 7 days ¶**	Yes / No
**20. Sorting the weaned piglets in the past 7 days ¶**	Yes / No
21. Sorting the fattener pigs in the past 7 days ¶	Yes / No
**22. Feeding sows in the past 7 days ¶**	Yes / No
23. Feeding suckling piglets in the past 7 days ¶	Yes / No
24. Feeding weaned piglets in the past 7 days ¶	Yes / No
25. Feeding fattener pigs in the past 7 days ¶	Yes / No
26. Washing sows in the past 7 days ¶	Yes / No
27. Washing suckling piglets in the past 7 days ¶	Yes / No
28. Washing weaned piglets in the past 7 days ¶	Yes / No
29. Washing fattener pigs in the past 7 days ¶	Yes / No
30. Healthcare tasks in sows in the past 7 days ¶	Yes / No
31. Healthcare tasks in suckling piglets in the past 7 days ¶	Yes / No
32. Healthcare tasks in weaned piglets in the past 7 days ¶	Yes / No
33. Healthcare tasks in fattener pigs in the past 7 days ¶	Yes / No
34. Birth assistance of sows in the past 7 days ¶	Yes / No

Question	Possible answers
35. Birth assistance of suckling piglets in the past 7 days ¶	Yes / No
36. Birth assistance of weaned piglets in the past 7 days ¶	Yes / No
37. Birth assistance of fattener pigs in the past 7 days ¶	Yes / No
38. Removing the manure of sows in the past 7 days ¶	Yes / No
39. Removing the manure of suckling piglets in the past 7 days ¶	Yes / No
40. Removing the manure of weaned piglets in the past 7 days ¶	Yes / No
41. Removing the manure of fattener pigs in the past 7 days ¶	Yes / No
42. Cleaning and disinfecting sows in the past 7 days ¶	Yes / No
43. Cleaning and disinfecting suckling piglets in the past 7 days ¶	Yes / No
**44. Cleaning and disinfecting weaned piglets in the past 7 days ¶**	Yes / No
45. Cleaning and disinfecting fattener pigs in the past 7 days ¶	Yes / No
46. Did you yourself administer antibiotics to the pigs in the last month? ¶	Yes / No
Personal hygiene:	
47. Do you use a mouth mask while working in the sheds? ¶	Yes / No
48. Do you wash your hands directly before entering the sheds? ¶	Yes / No
49. Do you wash your hands directly after leaving the sheds? ¶	Yes / No
Individual health condition:	
50. Were you hospitalized in or did you visit a hospital in the Netherlands in the last 6/12 months? ¶	Yes / No
51. Were you hospitalized in another Dutch health institution or did you visit one in the last 6/12 months?	Yes / No
52. Were you hospitalized in a foreign hospital or did you visit one in the last 6/12 months?	Yes / No
53. Have you received homecare in the last 6/12 months?	Yes / No
54. Did you visit your general practitioner in the last 6/12 months? ¶	Yes / No
55. Did you visit a specialist on the outpatient clinic in the last 6/12 months?	Yes / No
56. Have you had allergies in the last 6/12 months? ¶	Yes / No
57. Have you had eczema in the last 6/12 months? ¶	Yes / No
58. Have you had psoriasis in the last 6/12 months? ¶	Yes / No
59. Have you had impetigo in the last 6/12 months?	Yes / No
60. Have you had open wounds in the last 6/12 months? ¶	Yes / No
61. Have you had an abscess in the last 6/12 months?	Yes / No
62. Have you had a furuncle in the last 6/12 months?	Yes / No
63. Have you had a cold in the last 6/12 months?	Yes / No
64. Have you had laryngitis in the last 6/12 months?	Yes / No
65. Have you had inflammation of the ear in the last 6/12 months?	Yes / No
66. Have you had sinusitis in the last 6/12 months?	Yes / No
67. Have you had pneumonia in the last 6/12 months?	Yes / No
68. Have you had COPD in the last 6/12 months?	Yes / No
69. Have you had blood poisoning in the last 6/12 months?	Yes / No
70. Have you had arthritis in the last 6/12 months?	Yes / No
71. Have you had diabetes in the last 6/12 months?	Yes / No
72. Have you had surgery in the last 6/12 months?	Yes / No
73. Have you been fitted with a joint prosthesis in the last 6/12 months?	Yes / No
74. Have you had an external fixator in the last 6/12 months?	Yes / No
75. Have you had an IV in the last 6/12 months?	Yes / No
76. Have you had an stomach tube in the last 6/12 months?	Yes / No
77. Have you had an intestinal tube in the last 6/12 months?	Yes / No
78. Have you had an trachea fistula in the last 6/12 months?	Yes / No
79. Have you had artificial respiration in the last 6/12 months?	Yes / No
80. Have you had a bladder catheter in the last 6/12 months?	Yes / No
81. Have you had dialysis in the last 6/12 months?	Yes / No
82. Have you had acupuncture in the last 6/12 months?	Yes / No
83. Have you had medication with a needle in the last 6/12 months?	Yes / No
84. Are you currently treated with medication?	Yes / No
a. Name medication.	text
b. Indication	text
c. Since date	date
85. Have you ever smoked or do you currently smoke? ¶	Yes / No
Contact with other people	
86. Did persons living in a foreign country stay at your place in the last 6/12 months? ¶	Yes / No
87. In which country do these persons live?	Country
88. Did you go to another country for holidays?	Yes / No
89. If more than 6/12 months ago, when was the last time?	Date
90. If less than 6/12 months ago, when was the last time?	Date
Contact with meat	
91. How many times per week do you eat meat? ¶	No.
92. How many times per week do you have contact with unheated/unprocessed meat (for examples during cooking or packaging)? ¶	No.

Bold type indicates variables presented in table 4 with p≤0.2 in the longitudinal analysis with mixed models.
¶Variables with less than 10% missing values, at least 10% of farms in each category. Random intercept generalized linear mixed models were fitted for each variable by using a macro statement in SAS.

Technical Appendix Table 3. ORs for determinants of livestock-associated MRSA positivity in pooled pig samples (n = 1,054) from 32 farms (full multivariable generalized linear mixed model), the Netherlands, 2011-2013*

Variable	N¶	OR (95%CI)	P-value
Sampling moment			
0 months	262	0.89 (0.47-1.71)	**<0.001**
6 months	290	2.30 (1.27-4.15)	
12 months	259	1.99 (1.15-3.42)	
18 months	243	Ref.	
Age group			
Gilts	212	1.11 (0.66-1.85)	**<0.001**
Finishers	140	4.25 (2.37-7.59)	
Suckling piglets	212	3.99 (2.40-6.63)	
Weaned piglets	280	10.40 (6.22-17.38)	
Sows	210	Ref.	
External supply of gilts†			
Open	630	4.87 (1.24-19.16)	**0.023**
Closed	424	Ref.	
Water supply for animals			
Tap water	424	2.42 (0.81-7.25)	0.112
Private source	630	Ref.	
Separate medication pipe			
Yes	750	0.76 (0.31-1.90)	0.559
No	304	Ref.	
Carcass barrels cleaned and disinfected after emptied			
Yes	398	0.62 (0.30-1.31)	0.214
No	656	Ref.	
There is a delivery room for materials			
Yes	804	0.37 (0.15-0.90)	**0.028**
No	250	Ref.	
Injection of antimicrobials in piglets during the first week			
Yes	601	1.45 (0.77-2.73)	0.249
No	453	Ref.	
Tooth clipping in piglets			
Yes	416	1.83 (0.61-5.46)	0.280
No	638	Ref.	
Piglets and/or fatteners are vaccinated			
Yes	870	1.37 (0.59-3.16)	0.460
No	184	Ref.	
Needles renewed per compartment			
Yes	738	1.38 (0.68-2.78)	0.370
No	316	Ref.	
Sows in stable groups			
Yes	594	0.61 (0.31-1.18)	0.141
No	460	Ref.	
Hygiene in piglet compartment			
Disinfection	149	0.81 (0.24-2.69)	0.773
Soaking	270	1.54 (0.53-4.51)	
Soaking and disinfection	431	1.28 (0.52-4.51)	
None	204	Ref.	
Hygiene in the mating section			
Soaking and disinfection	169	0.50 (0.18-1.36)	0.173
None	885	Ref.	
Antimicrobial use per2-fold increase ($\log_2$ DDDA/Y)	1054	1.21 (1.00-1.45)	**0.049**
Use of cephalosporins			
Yes	84	2.76 (1.24-6.14)	**0.013**
No	970	Ref.	

Model fit: -2 Log RSPL= 5397,87

*Full multivariable model with random intercept for farm, sampling time and animal age group as terms of adjustment. All variables from the longitudinal risk factor analysis in pigs with p≤0.0.05 (table 5) are fitted in the model together with overall antimicrobial use ($\log_2$ DDDA/Y) and use of cephalosporins. MRSA, methicillin-resistant *Staphylococcus aureus*; OR, odds ratio; DDDA/Y, defined daily dosages animal per year; Ref, reference category; RSPL, residual pseudo-likelihood.

¶Multiple variables had missing values in the full model reducing the number of observations in the final model (table 6).

†Farms were defined as open when they received external supplies of gilts ≥1 time per year from at least 1 supplier and as closed when they received no external supply of gilts.
